# Effects of Prolonged Silver Nanoparticle Exposure on the Contextual Cognition and Behavior of Mammals

**DOI:** 10.3390/ma11040558

**Published:** 2018-04-05

**Authors:** Anna Antsiferova, Marina Kopaeva, Pavel Kashkarov

**Affiliations:** 1 National Research Center “Kurchatov Institute”, 1 Akademika Kurchatova sq., Moscow 123182, Russia; m.kopaeva@mail.ru (M.K.); kashkarov_pk@nrcki.ru (P.K.); 2 Moscow Institute of Physics and Technologies (State University), 9 Institutskiy Lane, Moscow Region, Dolgoprudny 141701, Russia; 3 Department of Physics, Lomonosov Moscow State University, 1-2 Leninskie Gory, Moscow 119991, Russia

**Keywords:** silver nanoparticles, behavioral functions, cognitive functions, mammals, open field, light-dark dox, elevated plus maze, contextual memory, accumulation in brain, neurotoxicity

## Abstract

Silver nanoparticles have been widely used in the lighting and food industries, in medicine, and in pharmaceutics as an antiseptic agent. Recent research demonstrates that, after prolonged oral administration, silver nanoparticles may cross the blood-brain barrier and accumulate in the brain in rather high amounts. In ex vivo experiments, it has also been shown that silver nanoparticles demonstrate neurotoxicity. The objective of this work was to answer the questions whether silver nanoparticles change cognitive and behavioral functions of mammals after prolonged administration if silver nanoparticles have accumulated in the brain. C57Bl/6 male mice were orally exposed to PVP-coated silver nanoparticles daily for 30, 60, 120 and 180 days. Control mice were exposed to distilled water. After that they were tested in the Open Field, Elevated Plus Maze, Light-Dark Box and contextual fear conditioning task. The data have shown that the experimental mice went through three periods of switching in the behavior caused by adaptation to the toxic silver nanoparticles: anxiety, appearance of research instinct and impairment of long-term memory. This provides evidence of the hazardous effect of silver nanoparticles, which appears after long periods of silver nanoparticle oral administration.

## 1. Introduction

Silver is a well-known antiseptic agent, which has been applied from ancient times. For instance, Hospitallers used silver dishes to protect themselves from bacterial and viral diseases [1]. Since the beginning of the 2000s, silver has been applied mostly in nanoform in food and light industries, medicine, pharmaceutics, etc. [2,3,4,5]. One type of application of nanosilver is in alternative medicine, where food supplements performed by solutions of silver nanoparticles (Ag-NPs) are recommended for humans for alteration of bacterial, viral and fungicidal diseases and to strengthen immunity. 

The antiseptic properties of silver and Ag-NPs are due to their toxicity [6]. One of the possible mechanisms of the toxicity is lysis, when silver ions accumulate in a bacterial cell and then the osmotic pressure tears the cell up. At the nanoscale level, silver possesses various properties such as high chemical activity and cellular penetrability, which make Ag-NPs more toxic than silver in its bulk form. 

Despite the increase of nanosilver’s positive antibacterial, antiviral and fungicidal properties, it can be toxic for healthy cells as well. This was shown in many in vitro and in vivo studies [6,7,8,9,10]. Recently, in in-vitro experiments, it was demonstrated that silver nanoparticles may cross the blood-brain barrier (BBB) of mammals, which protects the brain from toxic substances, and accumulate there [11,12]. Moreover, Ag-NPs are difficult to eliminate from the brain, which may be due to the low exocytosis level and low number of immune cells [13,14]. For instance, Ag-NPs do not tend to accumulate in organs with a high number of immune cells such as liver and blood that can easily get rid of the Ag-NPs. PVP-coated Ag-NPs were found in rather high amounts in blood, liver and brain after 2 months of daily oral administration of the nanoparticles (NPs) but after 1 month of washing them up with distilled water, 75% and 80% of the silver was eliminated from the liver and the blood, respectively, and just 5% of the silver was eliminated from the brain [13]. Further research on the investigation of Ag-NP biokinetics in mammals protractedly exposed to the NPs was performed in [14]. In this research, PVP-coated Ag-NPs were orally administrated to laboratory animals daily for 60, 120, 180 and 240 days in the amount of 100 µg of silver per day per mouse in order to study Ag-Np accumulation. Exposition was cancelled for the other groups of mammals after the pointed periods of the administration and silver was washed up with sterile water during 30 days for 60 days of administration, 60 days for 120 days of administration, 30, 60 and 90 days for 180 days of administration to study the elimination of silver. The content of silver in the brain and blood was measured by Instrumental Neutron Activation Analysis [15]. A gradual increase of silver in the brain over time was observed. The same could not be said about the blood. The rate of the elimination of silver from the blood was relatively high during the whole study and remained approximately constant. The rate of silver elimination from the brain was a time-dependent function. Firstly, it was slow but it drastically increased from 120 days of the administration. These findings indicated some negative processes in brain caused by prolonged exposure to Ag-NPs, which is important for further research.

The brain is composed of two major cell types, neurons and glial and also components of the brain vasculature including endothelial cells, smooth muscle cells, and pericytes [16]. Crossing BBB Ag-NPs may interact with all the cell types causing neurotoxicity. Firstly, they interact with astrocytes. It has been shown in ex vivo research that PVP-coated Ag-NPs were accumulated by rat astrocytes in a time- and dose- dependent manner but they did not affect cell viability [17]. Another research demonstrated the appearance of oxidative stress and acute calcium signals in primary astrocyte cultures in the ex vivo experiment [18]. 

Interaction with neurons requires a lot of attention because their impairment may cause dementia and neurodegenerative diseases. It was reported that Ag-NPs can induce significant cytotoxicity in cultured cerebral cortical neurons in a dose-dependent and time-dependent manner. The viability of cortical neurons significantly decreased at the tested time points after treatment with Ag-NPs at concentrations of 2 µg/mL. The results of this study suggested that Ag-NPs may induce the apoptosis of cortical neuronal cells by enhancement of intracellular ROS generation [19]. There are some other studies that prove the neurotoxicity of Ag-NPs by establishing this effect in ex vivo and in vitro experiments [20,21,22,23,24].

Histopathological data is emerging that summarizes the accumulation of Ag-NPs in the brain and their neurotoxicity. This constitutes the other branch of research such as a study of the influence of Ag-NPs on cognitive and behavioral functions. Some evidences of the slight ability and disability of the Ag-NPs to change brain functions has been made but all of them were established for rather shot periods of Ag-NP administration [25,26,27,28,29,30]. 

Rats were exposed to uncoated and PVP-coated Ag-NPs for 35 days and tested in the Morris Water Maze (MWM) [25]. Latent time was significantly higher for the rats exposed to uncoated NPs. However, no other drastic differences in behavior have been found. Another experiment [26] demonstrates that there is no effect on reference memory and working memory of Ag-NP in MWM after 7-day intraperitoneal injection of the NPs. However, 7-day treatment of Wistar rats with coated Ag-NPs significantly decreased spontaneous alternation in the Y-maze task and working memory functions in the radial arm-maze task, suggesting that nanoparticles have effects on short-term memory, without affecting the long-term memory [27]. It was supposed that the memory deficit is caused by the oxidative stress in the brain due to the Ag-NPs.

Prenatal exposure to Ag-NPs significantly impaired the cognitive behavior of mice in the MWM. Although there were no effects on the anxiety-like behaviors in the Elevated Plus Maze (EPM) in the treated offspring, the number of defecations and leanings in the Open Field test (OF) and number of passages in the Light-Dark Box test (LDB) were greater in the groups that were prenatally treated with Ag-NPs. Most of the impairments were more apparent in the offspring which had been prenatally exposed to high doses of Ag-NPs, particularly female ones [28]. Research [29] demonstrated a negative influence of the intranasal injection of Ag-NPs to mice in the MWM. Male rats were exposed to Ag-NPs for 1 week and several impairments of cognitive and motor functions have been found [30].

Taking into account the fact that humans face Ag-NPs daily in food, medicine, household goods, food supplements and from the environment in drinking water, due to the recycling of low degradable Ag-NPs, a lot of attention should be payed to the long-term Ag-NPs administration studies. Not only acute experiments but also prolonged studies should be considered. Therefore, the aim of the present research was to study the influence of Ag-NPs on mammal behavioral and cognitive functions after long-time administration of the NPs.

## 2. Materials and Methods

### 2.1. Nanoparticles

The colloidal solution of Ag-NPs performed by the food supplement Argovit C manufactured in the Russian Federation, Novosibirsk was used as nanosilver. It was packed in an opaque plastic 0.5 L bottle. The NPs were coated with PVP, the initial concentration of silver in the solution was 10 µg/mL in the distilled water. 

Dynamical Light Scattering (DLS) and Transmission Electron Microscopy (TEM) were used to measure the size and stability of the NPs. TEM has also been applied to visualize the NPs. For this purpose, the initial solution was dissolved by distilled water up to the concentration of 0.1 µg/mL in the amount of 15 mL. Half of the solution was used for the immediate measurements, the residual part has been preserved in the refrigerator at the temperature +2 °C for 1 year and then investigated with DLS and TEM to check the stability of Ag-NPs.

For the immediate measurements the solution was divided into two equal parts for DLS and for TEM. Before the measurements the solutions were sonicated in the ultrasonic bath for 15 min. For DLS, 1 mL of the solution per measurement was poured into a plastic cuvette and a series of measurements was accomplished. For TEM, 0.01 mL of the solution were applied to a carbon grid and dried, and microscopic measurements were made. The same experiment was accomplished with the preserved solution.

### 2.2. Animals

Eighty C57Вl/6 eight-week-old male mice, which weighed 19–27 g were purchased from the Stolbovaya supplier, Moscow, Russia. Mice were kept in individual ventilated cages with the access to standard laboratory food and water ad libitum under the controlled average room temperature of 23 ± 2 °C and a 12:12 h light/dark cycle. All experimental procedures were performed in accordance with the rules of the Ministry of Health of the Russian Federation (No. 267 of 19.06.2013) and the Local Ethics Committee for Biomedical Research of the National Research Center ‘Kurchatov Institute’.

Animals were randomly divided to four experimental groups with 20 mice in each group: “30 days”, “60 days”, “120 days” and “180 days”. One part of the animals in each group (n = 10) orally received Ag-NP suspended in distilled water (50 µg per day) daily during the whole experiment. The amount of consumed NPs was controlled by daily changing of the solution, cleaning the inner surface of the drink containers and weighing the filled drinking containers before and after each day of the exposure, calculating the amount of the solution that was drunk. The conditions with the certain humidity and temperature maintained on average the same amount of consumed solution, i.e., NPs during the experiment. The remaining part (control, n = 10) received sterile water.

Body weight was monitored weekly during the whole exposure period.

Behavioral functions of the mice including locomotor activity, research activity and anxiety-like behavior were assessed by commonly used neurobehavioral paradigms and the results were compared according to the treatment.

### 2.3. Open Field (OF)

Mice of different groups were tested in the OF on the 31st (or 61st, 121st and 181st respectively) day of the experiment. The OF is a plastic circular arena (d = 120 cm, h = 45 cm). Animals were placed in the center of the arena. Mice were allowed to freely explore the OF for 5 min. Following the experimental session, the mice were carefully removed from the OF, and returned to their home cages. The arena was cleaned with 70% ethanol between the subjects. The trajectories of animal movements were recorded using an automated video tracking system consisting of a Sony video camera (Tokyo, Japan) located 2.5 m above the arena and a behavior video recording system EthoVision XT 8.5 (Noldus Information Technology, Wageningen, The Netherlands). The obtained videos were analyzed in the computer program EthoVision XT 8.5. The arena was subdivided into three areas: central (d = 60 cm), peripheral (r = 10 cm, near the wall) and intermediate (between central and peripheral). Animals were compared by the following parameters: distance traveled (total, central, intermediate and peripheral), average speed (overall and in the areas), time spent in the areas, central and intermediate areas entries, latent period of exit from the central area, number of rearings.

### 2.4. Elevated Plus Maze (EPM)

Anxiety-like behavior of mice was evaluated on the 33rd (or 63rd, 123rd and 183rd respectively) day of the experiment. The EPM is comprised of two open arms (30 × 5 × 0.5 cm^3^) and perpendicular two closed arms (30 × 5 × 15 cm^3^), which are connected by a common 5 × 5 cm^2^ central square. The entire apparatus is 70 cm above the floor. Mice were placed in the central square, facing an open arm and were allowed to explore the apparatus for 5 min. The maze was cleaned with 70% ethanol after each test. The trajectories of animal movements were recorded using an automated video tracking system consisting of a Sony video camera (Japan) located 2 m above the maze and a behavior video recording system EthoVision XT 8.5 (Noldus Information Technology, The Netherlands). The obtained videos were analyzed in the computer program EthoVision XT 8.5.

Animals were compared by the following parameters: open and closed arms entries, open and closed arm duration, head dipping, rearing, latent period of the first approach in the closed and the open arms, total distance traveled, overall average velocity.

### 2.5. Light-Dark Box (LDB)

Anxiety-like behavior and stress-like behavior of mice were evaluated on 35th (or 65th, 125th and 185th respectively) day of the experiment. The LDB test is based on the innate aversion of rodents from illuminated areas and, on their spontaneous exploratory behavior in response to mild stressors, that is, novel environment and light. The apparatus consists of two light and dark plexiglass chambers (50 × 25 × 40 cm^3^) which are connected to each other by a 8 × 6 opening, and was cleaned with 70% ethanol after each test. Each mouse was placed individually in the middle of the light chamber. Mice were allowed to move freely between the two chambers and recordings were made over a 10-min period. A video tracking device captured the movements of the mice in the light chamber by a Sony video camera (Japan) located 2 m above the box and video recording system EthoVision XT 8.5.

Animals were compared by the following parameters: distance traveled and average speed in the light chamber, the time spent in the light chamber, the latency to cross to the dark chamber, the total number of transitions through the opening and the number of times that they peeped out of the dark compartment. 

### 2.6. Contextual Fear Conditioning

To test the effect of silver nanoparticles on memory formation and retention, mice were trained and their memory was tested in a contextual fear conditioning task using a Video Fear Conditioning System (MED Associates Inc., Fairfax, VT) and the computer program Video Freeze v2.5.5.0 (MED Associates Inc.). Video recordings of the animal behavior were made during training (on 48th, 78th, 138th and 198th day, respectively) and testing (on 49th, 79th, 139th and 199th day, respectively). The number and duration of freezing acts were determined automatically.

Animals of each experimental group (the AgNPs exposed mice and control mice separately) were randomly assigned to two subgroups: “active control (AC)” (n = 4) and “fear conditioning (FC)” (n = 6). Mice from the “AC” subgroup were placed for 6 min into the experimental camera, where they freely explored a new environment without a foot shock. Mice from ‘FC’ subgroup were placed for 6 min into the experimental camera, where they freely explored a new environment for 3 min, and then 3 foot shocks (1 mA, 2 s) were delivered with a 1 min interval followed by a 1 min rest period. Mice were returned to their home cages immediately after training. Twenty-four hours after training, animals were tested for long-term memory retention (3 min in the experimental camera without a foot shock). The proportion of freezing acts versus test duration was assessed as a measure of long-term memory. Before placing each animal into the chamber, the chamber was wiped with 70% ethanol.

Animals were compared by the following parameters: the percentage of freezing acts in the range before and after the current supply during training, the percentage of freezing acts during testing.

### 2.7. Bodyweight and Organ Weight

Bodyweight was controlled every week and the organ weight (brain, heart, kidneys, liver, spleen and lungs) of 6 mice that were selected randomly from each group was measured at the end of the study.

### 2.8. Statistical Analysis

Statistical analysis was performed with GraphPad Prizm 6 (La Jolla, CA, USA) by the nonparametric Mann-Whitney test. The differences were considered significant at *p* < 0.05. All data were expressed as means ± SEM.

## 3. Results

### 3.1. Nanoparticles

**Figure 1:**
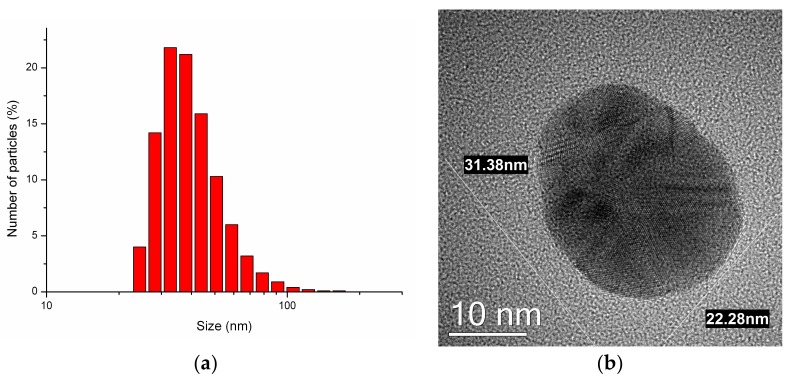
(**a**) Distribution of a number of particles by size for the initial measurements with Argovit C delivered with Dynamical Light Scattering (DLS); (**b**) Microphotograph of Argovit C silver nanoparticle Ag-NP visualized by Transmission Electron Microscopy (TEM).

In the immediate DLS and TEM experiments, the average size of Ag-NPs was 34 ± 2 nm (Figure 1a) and 31 ± 10 nm respectively. The TEM data showed that NPs were quasi-spherical (Figure 1b). 

The size of NPs that were preserved for 1 year due to DLS and TEM was 36 ± 6 nm and 32 ± 11 nm respectively. The visualization did not show significant agglomeration. Summing up all the evaluated data on the NP size, the high stability of Argovit C Ag-NPs was shown. 

### 3.2. Bodyweight and Organ Weight

**Figure 2:**
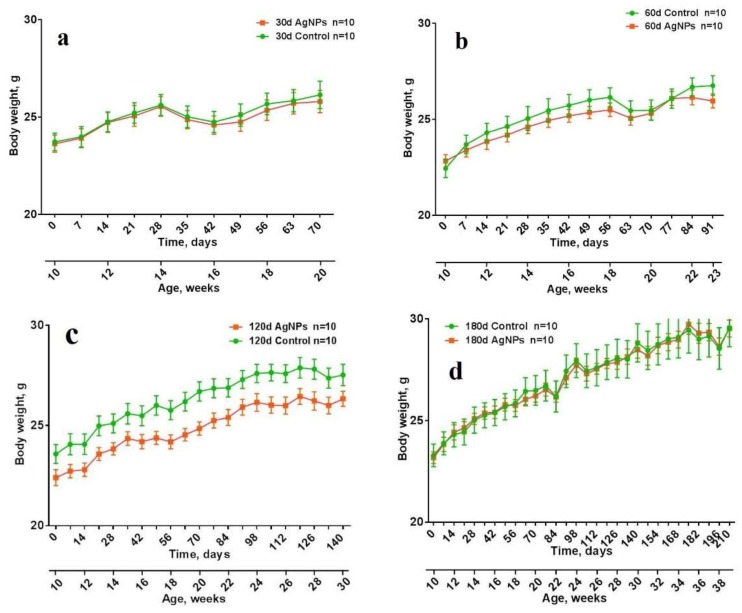
Dependence of the mice body weight on time: “30 days” (**a**); “60 days” (**b**); “120 days” (**c**); “180 days” (**d**).

Figure 2a–d shows that the mice grew normally during the experiment and there was no difference between the experimental and reference mice except for the “120 days” (Figure 2c) mice, where the Ag-NP group grew slower during whole period of the experiment. This may be caused by the individual characteristics of the mice in this group. However, some loss of wait can be observed for the testing periods and interestingly, the same oscillations in the body weight were observed for “120 days” and “180 days” (Figure 2c,d) mice that correlate with the testing of the “30 days”, “60 days” and “120 days” mice. This can be explained by the fact that their chambers were close to each other and mice became anxious because they saw that the other mice disappeared or it might be due to non-verbal communications. 

**Figure 3:**
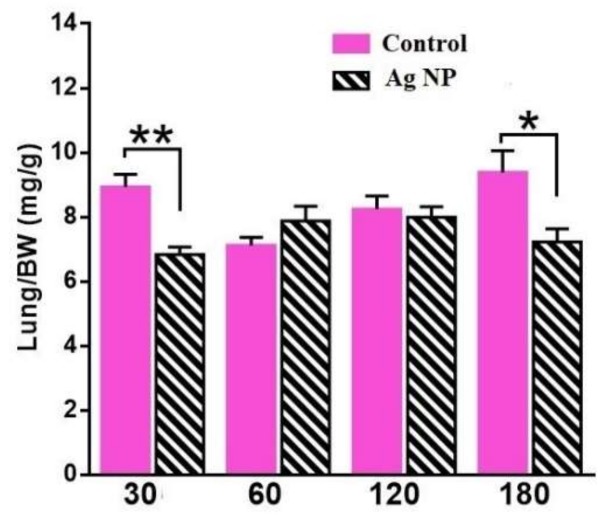
Weight of lungs per body weight for all groups. Reliable difference can be seen for the “30 days” and for the “180 days”.

Figure 3 demonstrates lung weight for the different periods of Ag-NP administration. Reliable differences between the reference and experimental groups were only observed for lungs. Ag-NP group lungs for 30 and 180 days weigh less than the reference ones (Figure 3).

### 3.3. Open Field

**Figure 4:**
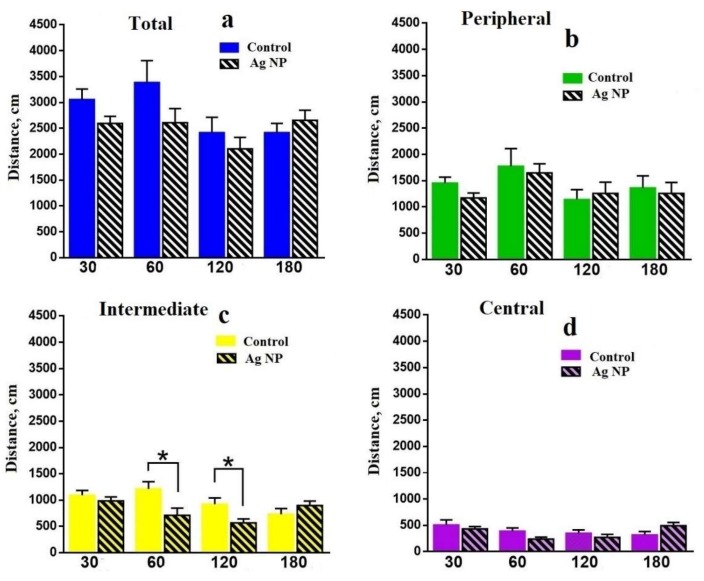
Distance traveled in the areas of the Open Field (OF): total distance (**a**); in the Peripheral area (**b**); in the Intermediate area (**c**); in the Central area (**d**).

Further, the data obtained in the OF is presented. Figure 4 shows that the locomotor activity of the mice in terms of distance moved in different areas of the OF. There is no reliable difference in the total moved distance (Figure 4a); however, the tendency shows that for all periods, reference mice moved longer distances, except the “180 days” group, where the situation was the reverse. A reliable difference can be seen in the Intermediate area for 60 and 120 days (Figure 4c), when the experimental mice moved a shorter distance. However, the tendency for 30 days is the same for all areas. The reverse tendency can be seen for 120 days in the Peripheral area (Figure 4b) and for 180 days in the Intermediate (Figure 4c) and Central (Figure 4d) areas, where the distance moved by experimental mice is higher, but for the Peripheral it is lower (Figure 4b). 

**Figure 5:**
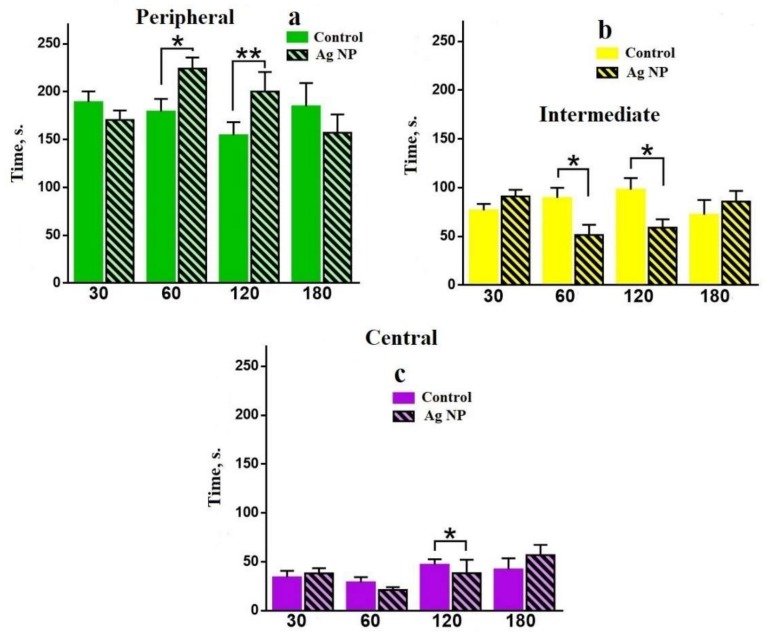
Time spent in the Peripheral (**a**); the Intermediate (**b**) and the Central (**c**) areas for different periods of the experiment.

Figure 5 demonstrates time spent in all areas of the OF. Firstly, for 30 days the tendency to avoid the Peripheral area and to choose Intermediate and Central areas for the experimental mice can be seen. Later, this tendency changes to reverse reliable difference: experimental mice choose the Peripheral area (Figure 5a) and avoided the Intermediate (Figure 5b) and the Central (Figure 5c) areas for 60 and 120 days. However, the tendency reversibly changed for 180 days, when the experimental mice chose the Intermediate and the Central areas and avoided the Peripheral area. 

**Figure 6:**
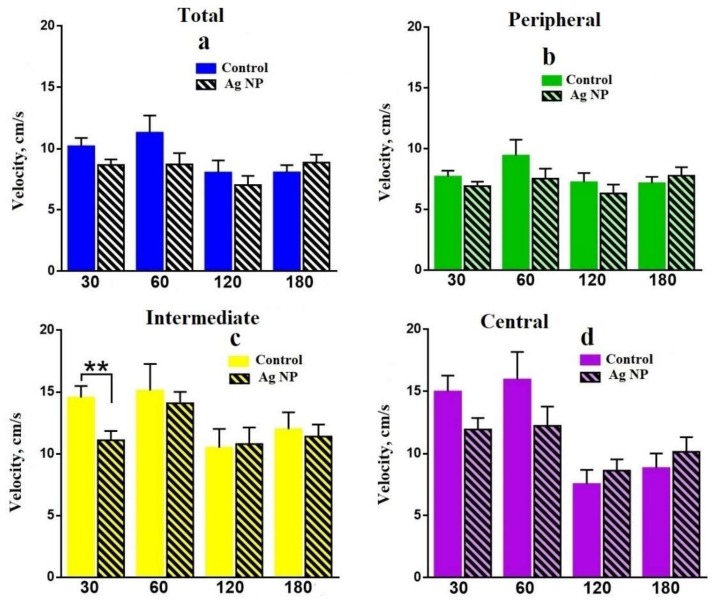
Mean velocity: total (**a**); in the Peripheral area (**b**); in the Intermediate area (**c**); in the Central area (**d**).

Some intermediate conclusions on the data obtained in the OF can be made. In absolute values, all groups of mice moved a longer distance and spent more time in the Peripheral area, followed by the Intermediate area. The least distance and time can be seen in the Central area, which is normal for mice. However, differences between the experimental and the reference mice demonstrate the influence of Ag-NPs on mice behavior. First of all, let us trace the reference mice. Slow degradation of the research instinct and the interest to explore the media performed by decreasing the traveled distance in more dangerous areas such as the Central and the Intermediate and enhancement of the time spent in all areas can be seen for all reference groups. This is due to aging. Mean velocity performed on the Figure 6 proves this assumption. Mice become slower with age.

Experimental mice demonstrated a different type of behavior up to 120 days of the experiment. Choosing the Peripheral area at the early and the intermediate stages of the experiment may be evidence of the appearance of anxiety which switched to reliably similar behavior with the control mice by 180 days.

**Figure 7:**
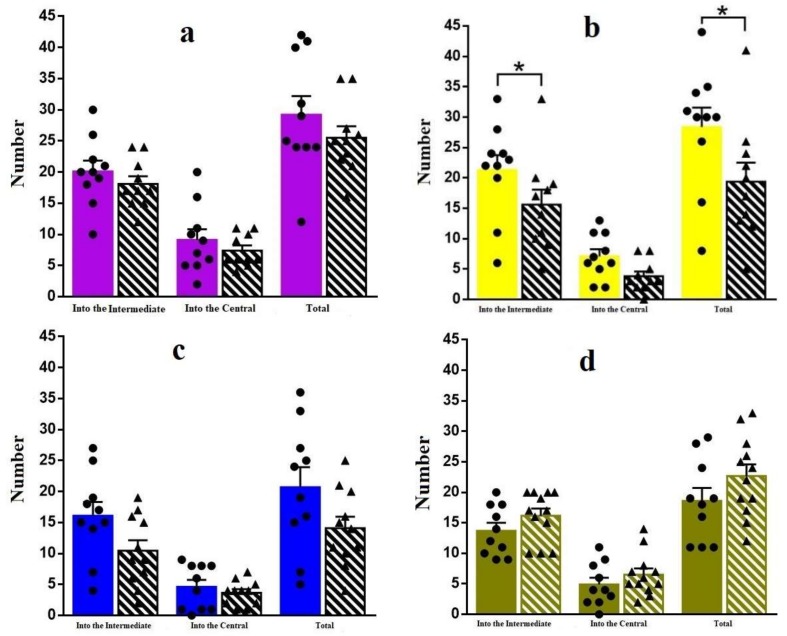
Number of transitions between the areas for different periods of Ag-NP administration: 30 days (**a**); 60 days (**b**); 120 days (**c**); 180 days (**d**).

Let us consider further behavioral characteristics in the OF. Figure 7 shows the number of transitions between different areas for different periods of the experiment. It can be seen that the number of transitions into the Central area is lower than the same value for the Intermediate area, which is normal for cautious mice behavior. Firstly, for 30 days (Figure 7a) only the tendency of transition number decreasing for Ag-NP group is observed. Reliable difference has been found for 60 days (Figure 7b), when the total number and the number of transitions into the Intermediate area for the experimental group is less than that of the reference group. The same tendency can be seen for the Central area as well. However, interestingly, the tendency changed reversibly for 180 days (Figure 7d). This could be due to the loss of cautions or to the research instinct development.

**Figure 8:**
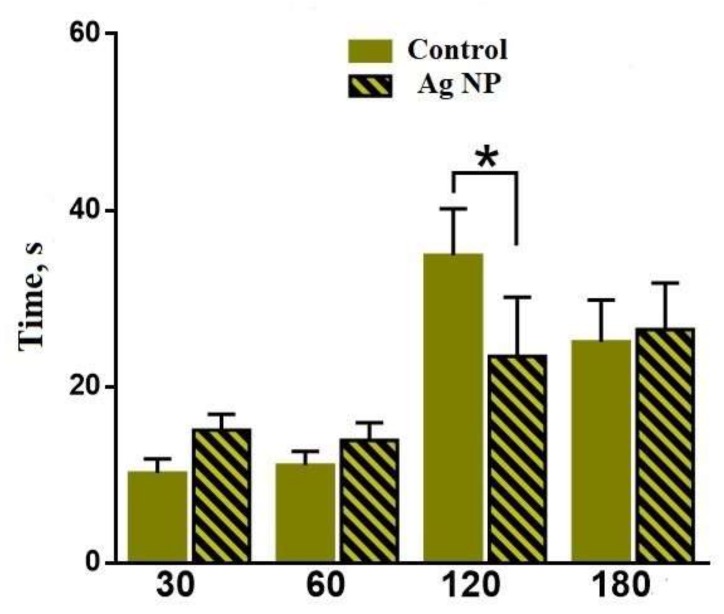
Latent time for different periods of the experiment. The reliable difference can be seen for 120 days.

Latent time (Figure 8) is the period of time between placing a mouse into the Central area and the first entrance into the Intermediate area. A reliable difference has been found for 120 days. The latent time for the Ag-NP mice is lower than for the reference mice, which could constitute evidence about the development of anxiety or the research instinct.

**Figure 9:**
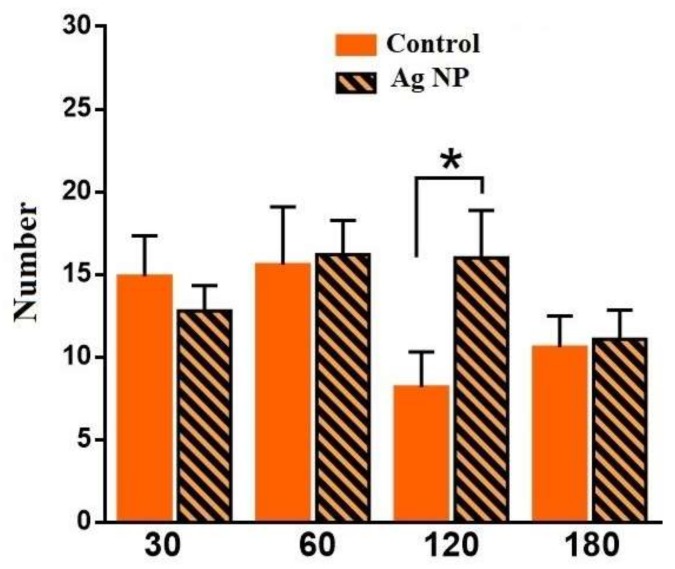
Number of rearings for different periods of the experiment. The reliable difference can be seen for 120 days.

Figure 9 demonstrates the number of rearings, which is typical behavior of mouse when it explores such media. It can be seen that for 120 days, Ag-NP mice made a reliably higher number of rearings. Taking into the account the number of transitions between areas and difference in latent time, it can be concluded that, up to 120 days, mice started to research the media more thoroughly. 

### 3.4. Elevated Plus Maze

Analyses of the data obtained from the EPM showed that Ag-NPs had no significant effects on the behavior of mice in EPM.

### 3.5. Light-Dark Box 

**Figure 10:**
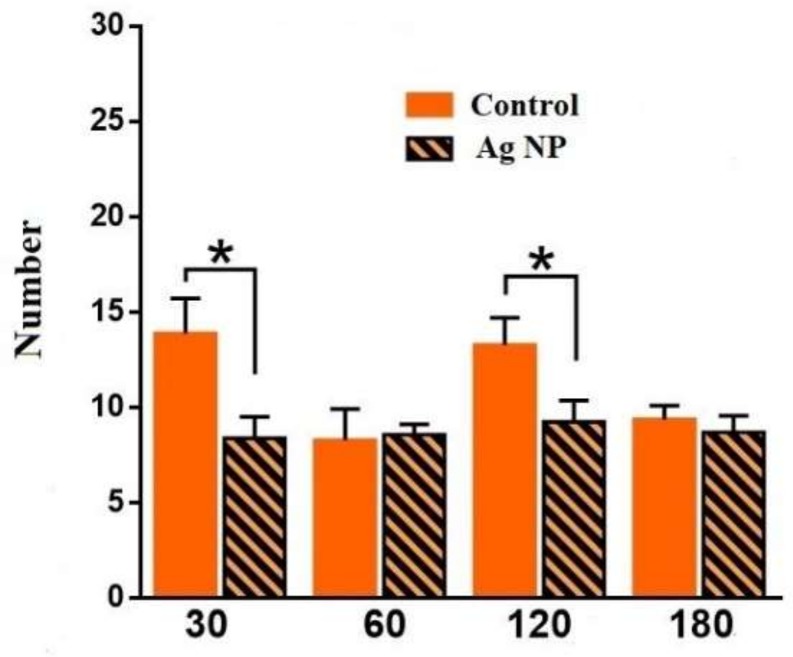
Number of times that mice peeped out of the dark chamber into the light chamber for the different periods of the experiment.

**Figure 11:**
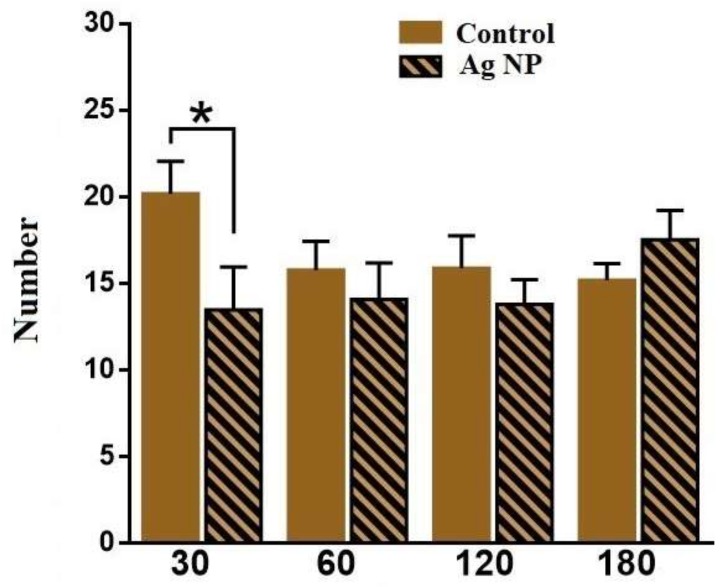
Number of transitions between the dark and the light chambers for the different periods of the experiment.

The following performed data are obtained in the LDB. Figure 10 demonstrates the number of times that mice peeped out of the dark chamber. The number is reliably lower for 30 and for 120 days, which may provide evidence about the mice’s anxiety. However, Figure 11 demonstrates the number of transitions between the chambers, which proves the proposal only for 30 days. For 120 days, no reliable difference in the number of transitions between the experimental and the control groups was observed. However, the tendency shows that the number of transitions for 180 days is even higher for the Ag-NP group. This result may evidence the presence of prevalence of research instinct for the experimental mice for the longer periods of Ag-NP administration than 30 days and some anxiety for 30 days. It also may show a decreasing caution in the Ag-NP mice when the research activity increased.

### 3.6. Contextual Fear Conditioning

**Figure 12:**
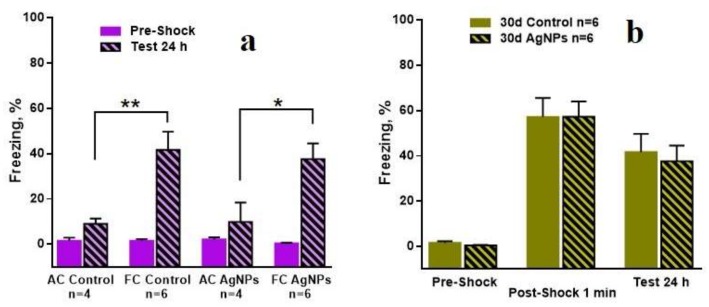
Number of the freezing acts in the Freezing chamber for “30 day” group of mice. (**a**) demonstrates the quality of learning for the Ag-NP groups and the reference groups between the fear conditioning (FC) and the active control (AC); (**b**) is the comparison in the learning and the formed contextual memory between the Ag-NP groups and the reference groups.

The most interesting results were received in the contextual fear conditioning model. Figure 12a shows the number of freezing acts for all “30 day” groups before learning and after learning. The numbers of the freezing acts before learning are low and reliably equal for all groups. The numbers of freezing acts are reliably higher for the FC of the control mice and for Ag-NP mice than for the AC at 24 h after learning. Figure 12b demonstrates the comparison in the freezing acts between the reference and the experimental groups for different periods of the test for the 30-day group. Firstly, it can be seen that the number of freezing acts for the Ag-NP and the reference mice is low before the electric pulse exposure. Then, 1 min after the exposure, the numbers of freezing acts dramatically increase for both groups and are reliably equal, which demonstrates the fine quality of learning. The numbers of the freezing acts in 24 hours after learning are slightly decreased for both groups and no difference can reliably be seen, which constitutes evidence of no contextual memory impairment.

Qualitatively similar data are obtained for “60 days” and for “120 days”; therefore, the results are not presented. 

**Figure 13:**
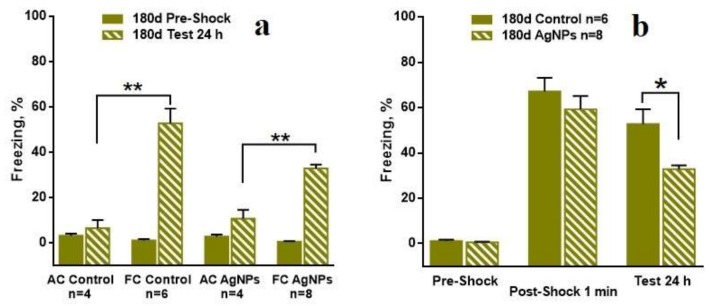
Number of the freezing acts in the freezing chamber for “180 day” group of mice. (**a**) demonstrates the quality of learning for the Ag-NP groups and the reference groups between the FC and the AC; (**b**) is the comparison in the learning and the formed contextual memory between Ag-NP groups and the reference groups. Reliable degradation of the contextual memory for Ag-NP group can be seen for the testing at 24 h after the exposure, which is performed by the number of freezing act’s decreasing.

The data of high interest were received for the “180 days” group, which is presented in Figure 13. Figure 13a shows the fine quality of learning performed by comparison between the AC and the FC groups within the Ag-NP and reference groups. The comparison between the Ag-NP groups and the reference groups led to an untypical result. Despite the good learning performed by the data on the number of the freezing acts in 1 min after the electric pulses exposure, the contextual memory testing in 24 h after the exposure demonstrates reliable decreasing of the freezing act number for the Ag-NP group (Figure 13b), which is evidence of a degradation of cognitive function.

## 4. Discussion

The data obtained in the behavioral tests such as the OF, the LDB and the EPM in the combination with the data received in the cognitive test provide evidence about the certain dependency in the influence of Ag-NPs on mammal cognitive functions. First of all, the anxiety in behavior, which can be regarded as a negative symptom, becomes noticeable for the periods of 30–60 days of the Ag-NP administration. Later, the anxiety is switched to the appearance of research instinct; the fear is lost and some improvement in behavior can be observed. However, for 180 days of the experiment the tendency of brave behavior was found. This could be regarded as a positive symptom; however, consideration of the data obtained in the cognitive test provides evidence about the reliable degradation of the contextual memory, which is obviously negative. A precise look at the data obtained in the behavioral tests shows that at the 180 days Ag-NP mice become slower and investigate the media more thoroughly, which is due to the loss of memory. However, no reliable results were found in the behavioral test and the drastic difference found in the cognitive test in the “180 days” group may be explained by the time difference in testing in the behavioral tests and in the contextual fear conditioning task. Thus, more time had been left and higher dose of the Ag-NPs had been received by the mice undergoing the cognitive testing; therefore, the effect was more obvious.

The idea is that the switching in behavior is due to the development of the adaptation mechanism to the potential toxic substance, which led to the changes in behavior. However, in terms of the contextual memory degradation, it caused space orientation difficulties and required the mice to spend more time investigating the media. Thus, mice went through two periods of adaptation to the toxic AG-NPs: anxiety and development of research instinct, which did not succeed because the Ag-NPs finally led to the degradation of the long-term memory.

Summing up the data of the high accumulation level of Ag-NPs in the brain, it can be concluded that Ag-NPs may be considered as a hazardous agent, which is most dangerous after relatively short (30–60 days) and long periods of administration (more than 120 days). Therefore, a lot of attention should be payed to the oral adopting of Ag-NP food supplements and the drugs in humans. If the doses are quite low but the period of administration is long, certain, perhaps, irreversible consequences in the cognition and behavior may occur.
